# Supplementary figures and images for: On the move: spatial ecology and habitat use of red fox in the Trans-Himalayan cold desert
Source: PeerJ. 2022 Sep 15;10:e13967. doi: 10.7717/peerj.13967 (PMC9482768; doi:10.7717/peerj.13967)

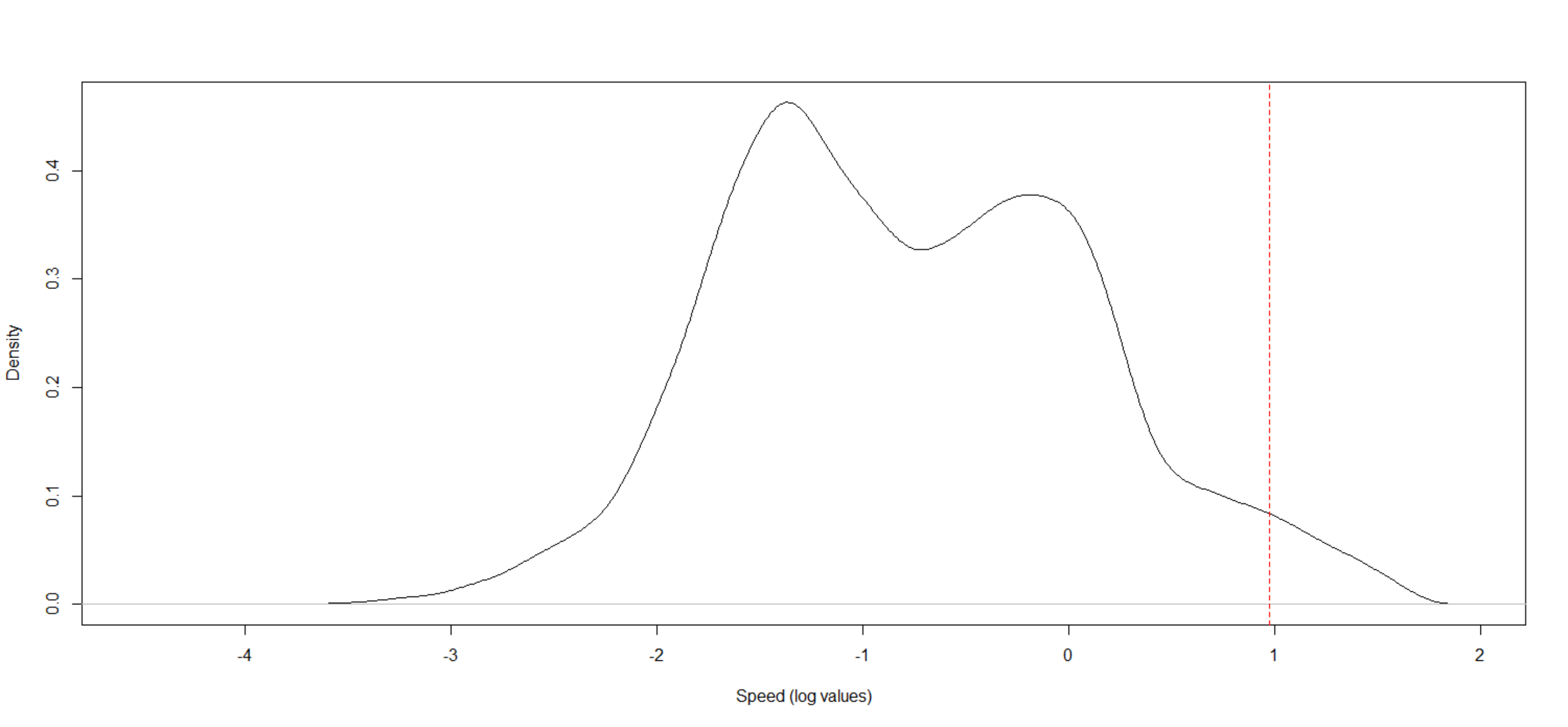

Supplement: Supplemental Information 1 [file peerj-10-13967-s001.png]

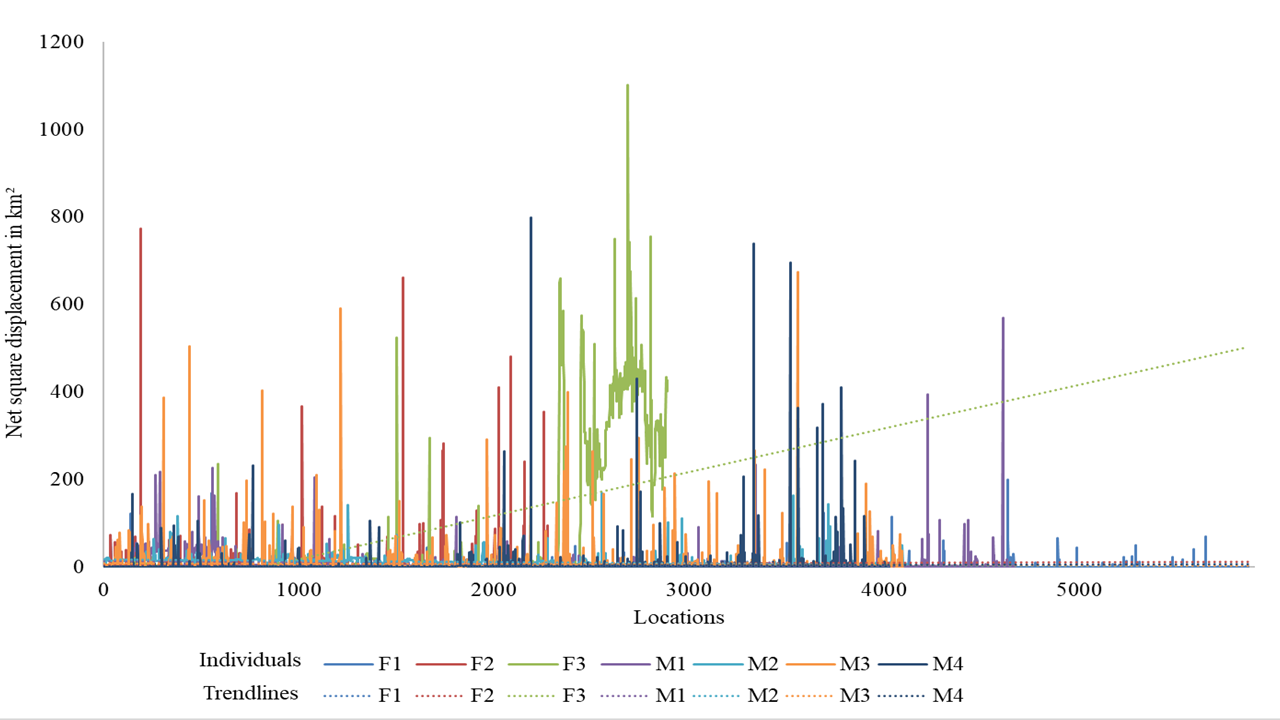

Supplement: Supplemental Information 2 [file peerj-10-13967-s002.png]

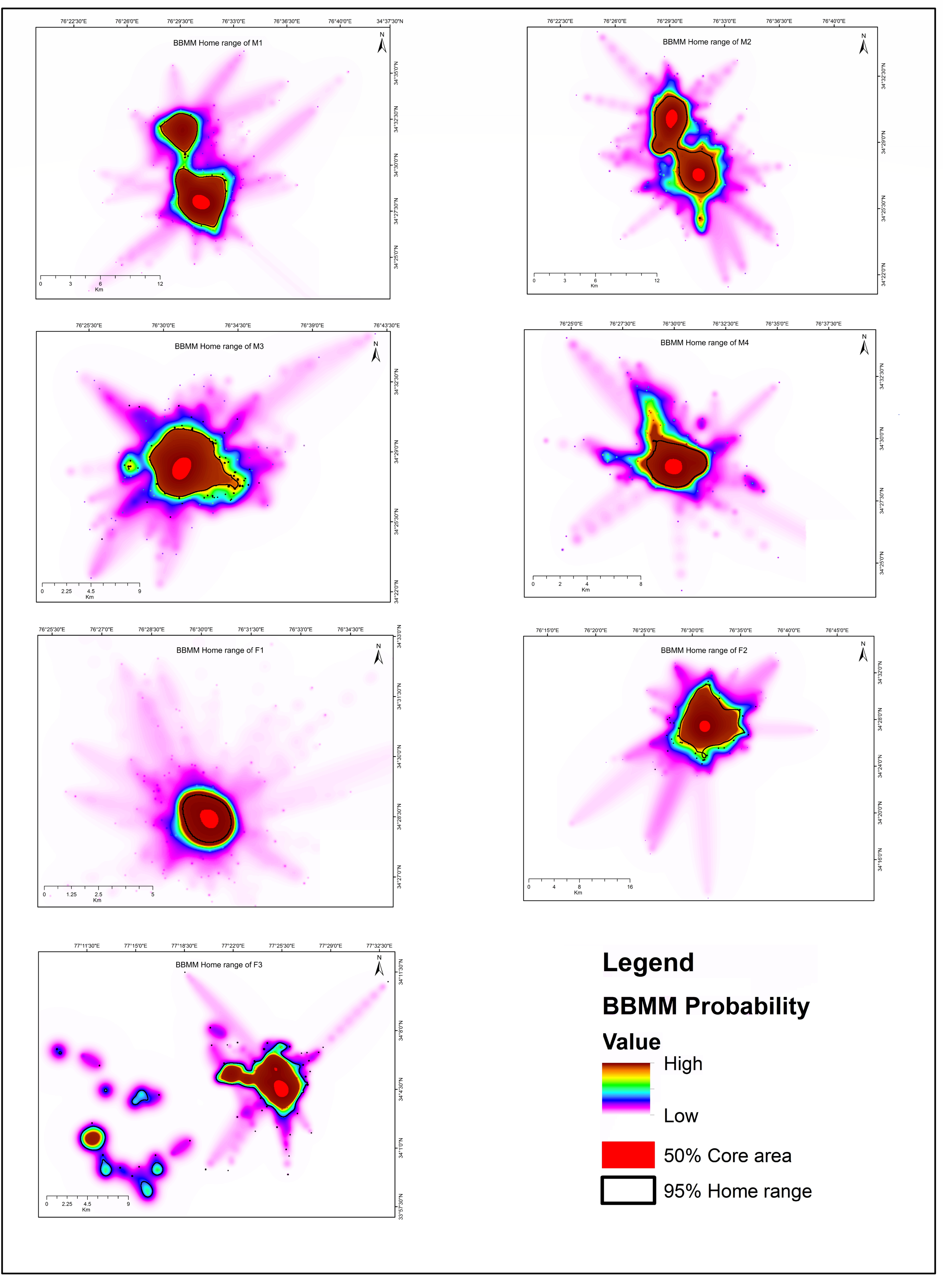

Supplement: Supplemental Information 3 [file peerj-10-13967-s003.png]
