# Supplementary material for: On the move: spatial ecology and habitat use of red fox in the Trans-Himalayan cold desert
Source: PeerJ. 2022 Sep 15;10:e13967. doi: 10.7717/peerj.13967 (PMC9482768; doi:10.7717/peerj.13967)
Supplement: Supplemental Information 4 [file peerj-10-13967-s004.docx]

*Table S1 Effect of down-sampling GPS fixes from 15-min to 2-h time interval on the estimated daily movement of red fox and t-test results*

| GPS Fixes | 15-min | 30-min | 1-h | 2-h | |
| --- | --- | --- | --- | --- | --- |
| Individuals  (No. of days) | Estimated daily average movements in km | | | | |
| F1 (90 d) | 11.10 | 8.62 | 6.10 | 4.52 | |
| F2 (56 d) | 20.92 | 18.81 | 15.57 | 12.33 | |
| F3 (79 d) | 14.72 | 13.24 | 11.99 | 8.42 | |
| M1 (83 d) | 11.61 | 8.83 | 6.35 | 3.38 | |
| M2 (75 d) | 10.91 | 8.17 | 5.62 | 3.72 | |
| M3 (30 d) | 34.22 | 29.40 | 20.67 | 13.60 | |
| M4 (30 d) | 20.85 | 17.66 | 13.27 | 9.75 | |
| Average (± SD) | 17.76 ± 8.45 | 14.96 ± 7.71 | 11.37 ± 5.68 | | 7.96 ± 4.18 |
| One tailed t-test | 15 min vs 30 min | 30 min vs 1h | 1 h vs 2h | | |
| p-value | < 0.001 | 0.004 | 0.001 | | |
